# Acetylcholine enhances deviance detection in Hodgkin-Huxley neuronal networks

**DOI:** 10.1007/s11571-026-10522-3

**Published:** 2026-08-07

**Authors:** Fei Fang, Zi-Gang Huang, Zenas C. Chao

**Affiliations:** 1 Key Laboratory of Biomedical Information Engineering of Ministry of Education, Institute of Health and Rehabilitation Science, School of Life Science and Technology, Xi’an Jiaotong University, Xi’an, 710049 China; 2 International Research Center for Neurointelligence (WPI-IRCN), UTIAS, The University of Tokyo, Tokyo, Japan

**Keywords:** Neuromodulator, Deviance detection, Hodgkin-Huxley, Acetylcholine

## Abstract

The brain’s ability to detect unexpected events, deviance detection (DD), is critical for survival. While DD has been computationally explained by synaptic plasticity, the role of neuromodulators such as acetylcholine (ACh) remains less understood. Here, we examine how ACh modulates DD. Using a cholinergic-sensitive Hodgkin-Huxley network of 200 neurons arranged in 2D space and stimulated via five spatially distinct inputs (A–E), we implemented an oddball paradigm with three conditions: standard (80% A, 20% B), deviant (20% A, 80% B), and a multi-standard control (20% each of A–E). ACh levels were modeled by varying the conductance of a slow K⁺ current, reflecting cholinergic modulation of the M-current. In the absence of ACh, the network already exhibited DD, responding more strongly to deviant A compared to control A. Notably, introducing a small amount of ACh amplified DD, while further increases suppressed it. Maximal DD occurred when strong spike frequency adaptation to standard B reduced competition, and enhanced phase-locking synchronized the network’s response to deviant A. These findings reveal how neuromodulation can shape context-sensitive neural computation, optimizing detection of salient events through a dynamic balance of suppression and synchronization.

**Supplementary Information:**

The online version contains supplementary material available at 10.1007/s11571-026-10522-3.

## Introduction

Context shapes how sensory information is processed in the mammalian brain. A well-known example is the enhanced neural response to contextually unexpected stimuli, known as deviance detection (DD). This phenomenon, observed even in early sensory cortices, reflects the brain’s ability to detect statistically improbable events or violations of regularities in the environment (Ishishita et al. 2019; Obara et al. 2023; Lao-Rodríguez et al. 2023; Parras et al. 2017; Tiitinen et al. 1994; Valerio et al. 2024; Yu et al. 2009; McCollum et al. 2024). Critically, DD involves two distinct components: stimulus-specific adaptation (SSA) and genuine deviance detection (genuine DD). SSA refers to reduced neural responses to frequently repeated stimuli, a form of repetition suppression that is specific to stimulus features (Hinz et al. 2025; Valerio et al. 2024; Yarden et al. 2022). While SSA can produce larger responses to rare stimuli, this alone does not confirm genuine DD. In contrast, genuine DD requires a stricter criterion: the neural response to a deviant stimulus must exceed the response to the same physical stimulus when it is not surprising (i.e., presented in a control condition). This distinction is essential for isolating the brain’s computation of contextual probability, rather than mere physical novelty.

The capacity for genuine DD is biologically and clinically significant. Impairments in DD have been reported as biomarkers in several neurodevelopmental and psychiatric disorders, including schizophrenia, autism, and attention deficit hyperactivity disorder (De Groote et al. 2021; Näätänen et al. 2014; Pérez-González et al. 2022), highlighting the need to understand its underlying mechanisms. Genuine DD has been documented across sensory modalities, including auditory, visual, and somatosensory systems (Azouz and Gray 2003; Bounds and Adesnik 2025; Derveer et al. 2023; McCollum et al. 2024; Obara et al. 2023; Parras et al. 2017; Wang et al. 2018), and across species ranging from rodents to humans (Behrens et al. 2009; Ulanovsky et al. 2004; Yarden and Nelken 2017). It has also been observed throughout the brain, from subcortical structures like the inferior colliculus to higher cortical areas (Chen et al. 2015; Tsolaki et al., 2015). Notably, recent work has demonstrated genuine DD in neuronal cell cultures (Zhang et al. 2025), showing that this computation can emerge from simple networks, independent of complex brain circuitry.

Computational models have typically attributed genuine DD to synaptic plasticity. While models based on long-term plasticity exist (Hertäg and Sprekeler 2020; Wacongne et al. 2012), the rapid emergence of DD in vivo points to fast-acting mechanisms. Short-term synaptic plasticity, particularly short-term depression, has been a primary candidate (Anwar et al. 2017; Mill et al. 2011; Yarden and Nelken 2017). More recent studies show that intrinsic neuronal adaptation, such as threshold adaptation, is also sufficient to generate genuine DD. Moreover, short-term depression and threshold adaptation can interact synergistically, enhancing DD beyond what either mechanism achieves alone (Kern and Chao 2023). These findings suggest that genuine DD can arise from the dynamic interaction of fast plasticity mechanisms, without requiring long-term structural changes.

Beyond synaptic and intrinsic mechanisms, neuromodulation also plays a key role in shaping neural responsiveness (Brzosko et al. 2019; Ghosh and Maunsell 2024; Sarter and Parikh 2005; Shine et al. 2021; Thiele and Bellgrove 2018; Wiseman et al. 2024; Yu and Dayan 2005). In particular ACh modulates neuronal excitability and synaptic transmission, and has been implicated in DD (Hasselmo and McGaughy 2004; Herrero et al. 2008; Kuczewski et al. 2005; Pérez-González et al. 2022, 2024; Picciotto et al. 2012). ACh enhances neural gain and modulates inhibition by acting on presynaptic interneurons (Colangelo et al. 2019). It also influences short-term synaptic plasticity via muscarinic and nicotinic receptors that regulate presynaptic transmitter release (McKay et al. 2007). Functionally, ACh increases the precision of bottom-up sensory transmission (Kang et al. 2014), which is essential for predictive coding, particularly in tuning the gain on prediction error signals (Pérez-González et al. 2024). Notably, ACh has been shown to modulate genuine DD. By employing a roving oddball paradigm and focusing analyses on the first presentation of the deviant stimulus, this effect was dissociated from subsequent sensory learning and adaptation (Moran et al. 2013). 

However, the mechanisms by which ACh contributes to genuine DD remain unclear. Existing computational studies of cholinergic modulation in Hodgkin-Huxley (HH) networks have primarily focused on how ACh alters neuronal excitability and synchronization (Roach et al. 2019), without examining its role in deviance detection. Likewise, computational models of genuine DD based on short-term plasticity have largely neglected neuromodulatory influences (Kern and Chao 2023). This omission is notable because experimental studies increasingly implicate ACh in deviance detection across multiple levels of the auditory system. Cholinergic signaling modulates deviance-sensitive responses throughout the auditory system, from the inferior colliculus to cortical circuits (Pérez-González et al. 2022). Within predictive coding frameworks, ACh has further been proposed to regulate the precision of prediction error signals rather than simply suppress responses to repeated stimuli (Pérez-González et al. 2024). Moreover, cholinergic degeneration in Alzheimer’s disease has been hypothesized to impair predictive coding and deviance detection, contributing to cognitive decline (Pérez-González et al. 2022). Together, these findings highlight the need for a mechanistic and computational understanding of how ACh shapes genuine DD.

To address this, we constructed a cholinergic-regulated HH network model devoid of other forms of synaptic plasticity. We systematically varied ACh levels across the network during a simulated oddball paradigm. Our results show that genuine DD is maximized at an intermediate level of ACh. Mechanistically, this effect arises from two complementary processes: local suppression via ACh-induced spike frequency adaptation (SFA), which frees up neural resources for deviant detection, and global synchronization via ACh-induced phase locking, which amplifies the network’s response to the deviant stimulus.

## Results

### A 2D network of cholinergic Hodgkin-Huxley spiking neurons

We used a HH-based model of cholinergic modulation in pyramidal cells to simulate neuron membrane potential dynamics (Hodgkin and Huxley 1952; Roach et al. 2019). Cholinergic signaling through M1 muscarinic ACh receptors can be well modeled by parameterizing the maximal conductance $\:{g}_{Ks}$ of a slow, low-threshold potassium-mediated adaptation current (see details in Methods).

**Fig. 1:**
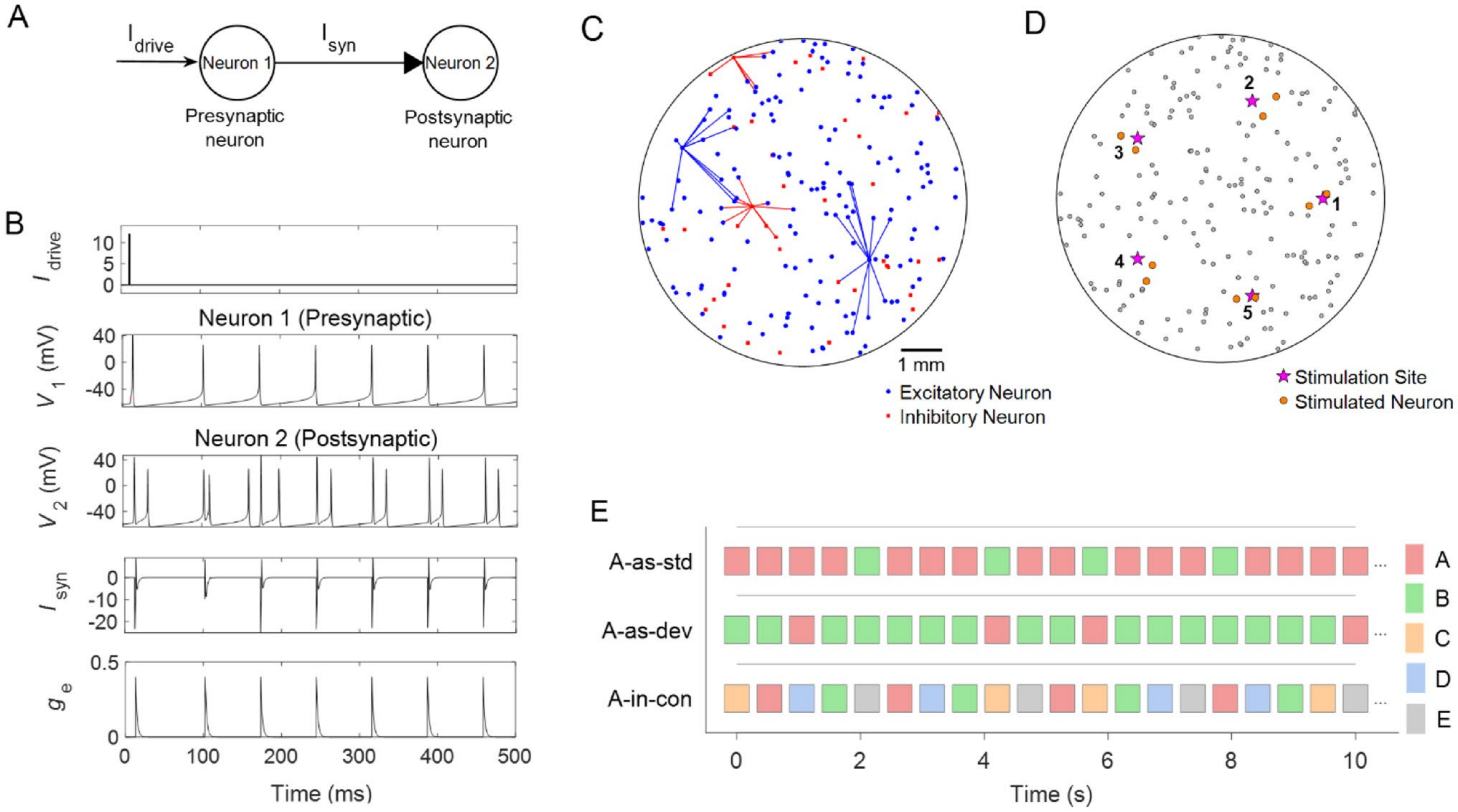
Neural network model and experimental setup. **A** Schematic of a two-neuron excitatory circuit. $\:{I}_{drive}$ and $\:{I}_{syn}$ represent external and synaptic currents, respectively. **B** Responses to a brief pulse of current stimulation. $\:{V}_{1}$ and $\:{V}_{2}$ denote the membrane potentials of Neurons 1 and 2 in panel A. The excitatory synaptic conductance is denoted as $\:{g}_{e}$. **C** Example network layout, illustrating the spatial locations of excitatory (red) and inhibitory (blue) neurons, along with all outgoing synaptic connections from two neurons of each type. **D** Stimulation design. Five stimulation sites (A–E) are evenly spaced on a circle with a 2.5 mm radius. When a site is stimulated, current is applied to the two neurons nearest to the electrode (highlighted in yellow). **E** Oddball stimulation paradigm. Each square represents a stimulation event, with the stimulus electrode indicated by color. Three sequence types are shown: A-as-std, A-as-dev, and A-in-con. Note that stimuli were delivered instantaneously (1 ms duration); the extended durations in the figure are for visualization only

To characterize the fundamental properties of synaptic transmission within our model, we first simulated a minimal two-neuron excitatory circuit (Fig. 1A). The presynaptic neuron (Neuron 1) was stimulated with a brief, suprathreshold current pulse ($\:{I}_{drive}$) at the beginning of the simulation. This single stimulus was sufficient to elicit a stable, periodic train of action potentials in Neuron 1 for the entire 500 ms recording period (Fig. 1B, $\:{V}_{1}$). The activity of Neuron 1 was effectively and reliably propagated to the postsynaptic neuron (Neuron 2). We observed a precise one-to-one firing relationship, where each action potential in the presynaptic neuron triggered a corresponding action potential in the postsynaptic neuron with a minimal delay (Fig. 1B, $\:{V}_{2}$). This faithful transmission was mediated by the dynamics of the excitatory synapse. Following each presynaptic spike, the postsynaptic excitatory conductance ($\:{g}_{e}$) was transiently activated, leading to a sharp, inward synaptic current ($\:{I}_{syn}$) in Neuron 2. These excitatory postsynaptic currents consistently depolarized the postsynaptic membrane to its firing threshold, thus ensuring robust signal transmission.

We constructed a network of 200 HH neurons (Fig. 1C), consisting of 160 excitatory (80%) and 40 inhibitory (20%) neurons. These neurons are randomly distributed within a two-dimensional circular area with a radius of 4 mm, resembling a neuronal cell culture (Zhang et al. 2025). Each neuron forms synapses with 10 randomly selected postsynaptic neurons within a distance of 2 mm for excitatory neurons and 1 mm for inhibitory neurons. The synaptic weight is set at w = 1.0 nS (see details in Methods). These specific parameters were chosen to ensure reliable responses to strong stimuli without inducing unstable or persistent activity. Connectivity and weights remain constant throughout the simulation. Stimulation is applied through five “virtual electrodes” (indicated by stars in Fig. 1D), evenly spaced at a distance of 2.5 mm from the center of the dish. These correspond to stimuli A, B, C, D, and E. Upon stimulation, current is injected into the three neurons (either excitatory or inhibitory) closest to the stimulus site (highlighted in yellow in Fig. 1D).

It is important to note that our goal is to isolate the effect of ACh; therefore, we use the simplest form of network dynamics, excluding both short-term plasticity (e.g., neuronal threshold adaptation or synaptic depression) and long-term plasticity (e.g., spike-timing-dependent plasticity).

### Oddball paradigm and simulation

To investigate genuine DD, we used a classical oddball paradigm (Fig. 1E), presenting a total of 200 stimuli at regular intervals of 100ms (see details in Methods). The target stimulus (labeled A) and distractor stimuli (labeled B, C, D, and E) were presented in three randomized sequences: (1) the standard sequence (A-as-std), consisting of 160 presentations of A and 40 of B; (2) the deviant sequence (A-as-dev), with 40 presentations of A and 160 of B; and (3) the many-standards control sequence (A-in-con), with 40 presentations of each stimulus (A–E). All stimuli were presented in random order. Each stimulus consisted of a 12 $\:\mu\:A/{cm}^{2}$ current pulse lasting 1 ms. In this design, stimulus A serves three roles: (1) as an expected stimulus in the standard sequence (A-as-std), (2) as an infrequent violation of the expected stimulus B in the deviant sequence (A-as-dev), and (3) as an equally infrequent stimulus in the many-standards control sequence (A-in-con), where no strong expectation is formed, allowing control for pure adaptation effects.

We constructed five networks with different structures. For each network, we applied the three stimulation sequences, each repeated four times with a different random stimulation order. A 1-second stabilization period was inserted between sequences to allow the network to fully recover before the next sequence began.

### Cholinergic effect on HH neuron and network

**Fig. 2:**
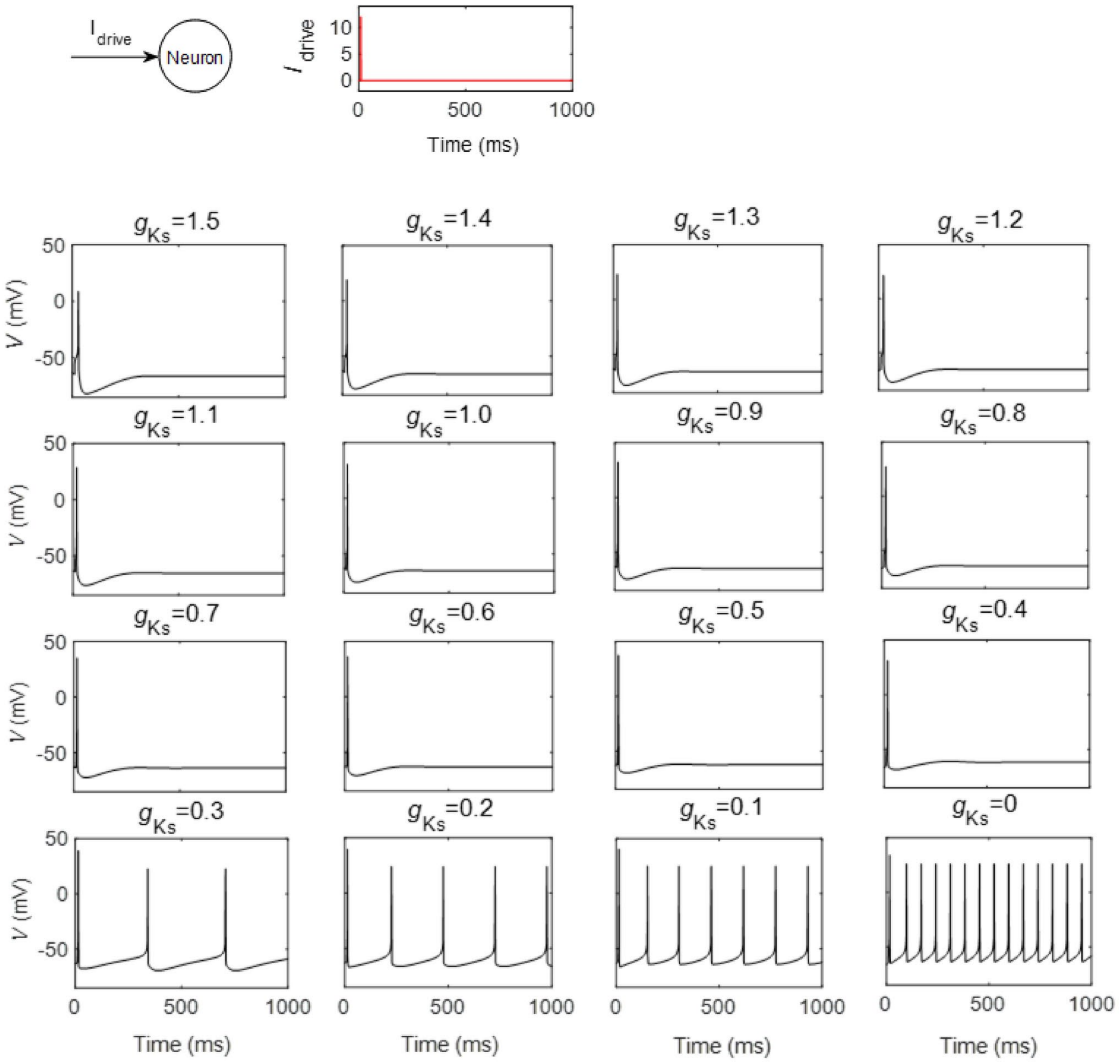
Effect of slow potassium conductance ($\:{g}_{Ks}$) on the firing pattern of a single HH neuron. Schematic of the setup: a single HH neuron receives a brief external current input ($\:{I}_{drive}$). Each panel shows the neuron’s membrane potential under a different $\:{g}_{Ks}$ value, ranging from 1.5 (corresponding to no ACh) to 0 (representing maximal ACh)

We first examined how the slow potassium current influences the firing properties of a single HH neuron by systematically varying $\:{g}_{Ks}$, the maximal conductance of the slow potassium current. As illustrated in Fig. 2, the neuron was subjected to a suprathreshold depolarizing current ($\:{I}_{drive}$) to elicit action potentials. When $\:{g}_{Ks}$ is high or intermediate (e.g., 1.5 to 0.4 mS/cm²), the neuron fires a single action potential, after which the membrane potential repolarizes and remains near the resting level (Fig. 2). As $\:{g}_{Ks}$ is progressively decreased, reflecting increasing levels of ACh, the firing pattern shifts. For $\:{g}_{Ks}$ $\:\le\:\:$0.3, the neuron transitions to a tonic firing mode, marked by sustained, rhythmic action potentials throughout the stimulus duration. These results demonstrate that the slow potassium current is a key factor for regulating the transition between phasic and tonic firing modes in single neurons.

**Fig. 3:**
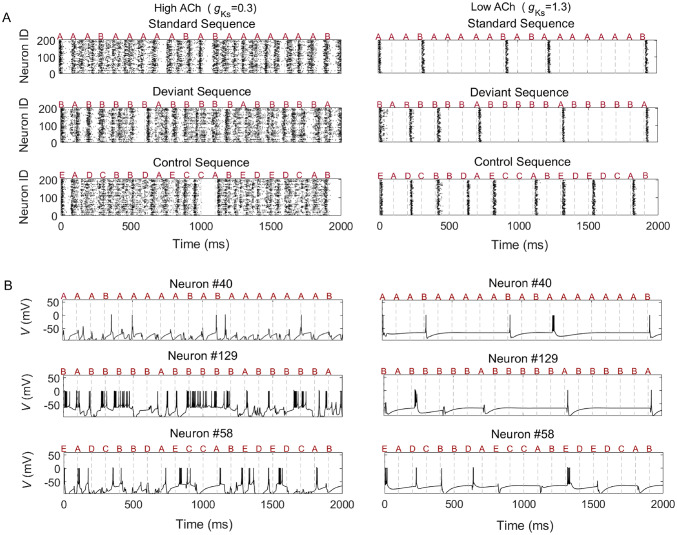
Network activity under different levels of $\:{g}_{Ks}$. **A** Raster plots showing the spiking activity of all 200 neurons in the network under high ACh (low $\:{g}_{Ks}$ = 0.3) and low ACh (high $\:{g}_{Ks}$ = 1.3) conditions. Different stimulation types (A–E) are indicated by red letters above the plot. Sequence types vary across rows. **B** Membrane potentials of three example neurons (#40, #129, and #58) from the network shown in panel **A**

Figure 3A shows the spiking responses of a sample network of 200 HH neurons to the three stimulation sequences under two levels of ACh. At $\:{g}_{Ks}$ = 0.3 (high ACh), the network exhibited strong but non-time-locked responses. In contrast, at $\:{g}_{Ks}$ = 1.3 (low ACh), evoked responses were less frequent but more tightly time-locked to the stimuli. Example membrane potentials of individual neurons are shown in Fig. 3B

### Cholinergic modulation of genuine DD

**Fig. 4:**
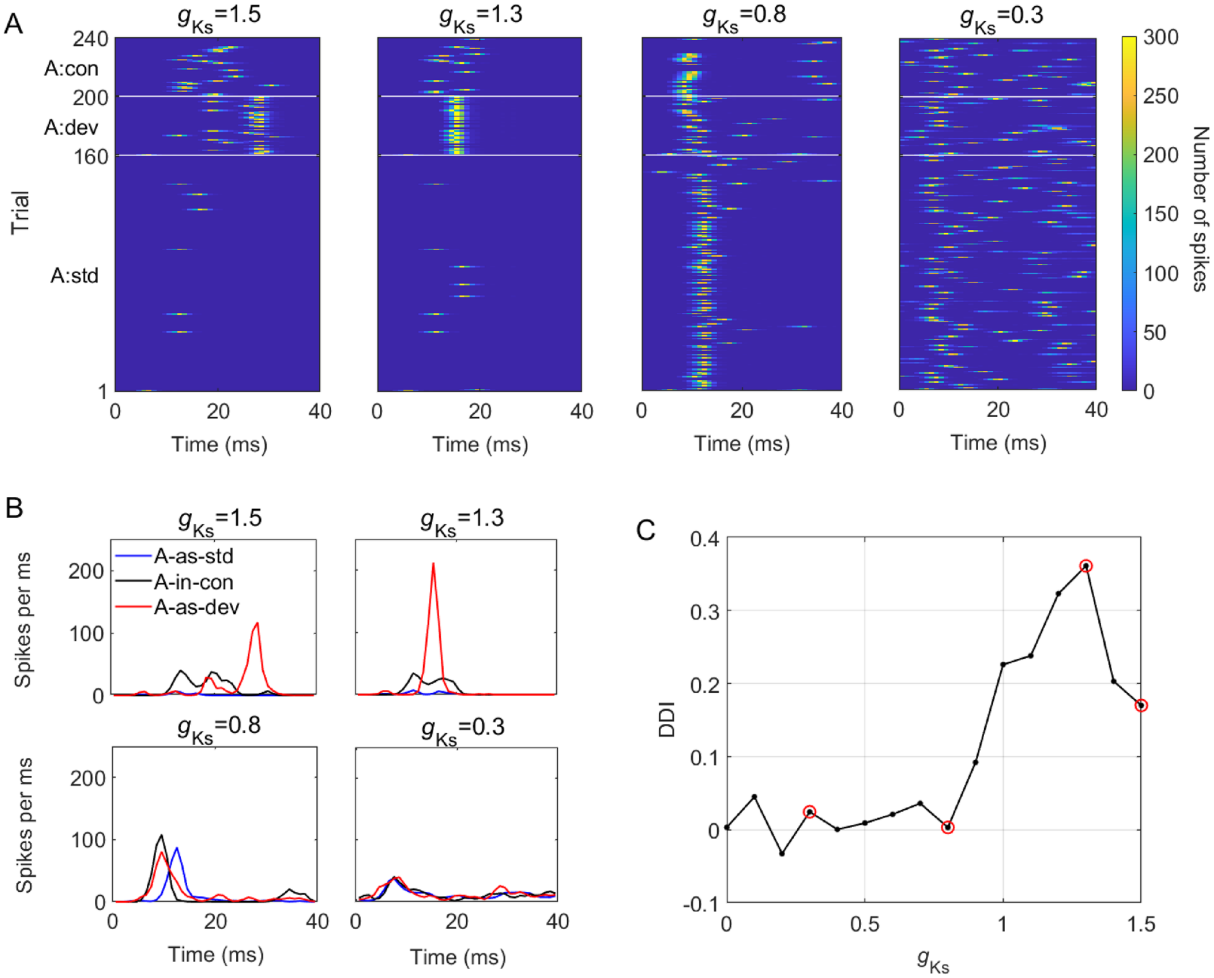
Deviance detection in a sample network under varying $\:{g}_{Ks}$ levels. **A** Network responses to the target stimulus A under three sequence types. A: std, A:dev, and A: con represent the evoked responses to stimulus A under the A-as-std (160 trials), A-as-dev (40 trials), and A-in-con (40 trials) sequences, respectively. Color indicates the total spike count across all neurons per millisecond. Four $\:{g}_{Ks}$ conditions (1.5, 1.3, 0.8, and 0.3) are shown. **B** Post-stimulus time histograms for network responses to stimulus A under the three sequence types. **C** DDI for each $\:{g}_{Ks}$ value in the sample network. The four examples shown in panels **A** and **B** are marked with red circles

To demonstrate that our model exhibits genuine DD, we first examined the evoked responses to stimulus A across all three sequences (A-as-dev, A-in-con, and A-as-std) in a sample network under four different $\:{g}_{Ks}$ values (1.5, 1.3, 0.8, and 0.3 mS/cm²) (Fig. 4). When $\:{g}_{Ks}$ = 1.5 (no ACh), stimulus A in the deviant sequence elicited a robust response with a latency of approximately 28.5 ms. In contrast, responses to A in the control sequence were less consistent, and in the standard sequence, only sporadic activity was observed. At $\:{g}_{Ks}$ = 1.3 (low ACh), strong evoked responses were still observed, but the latency shifted earlier to around 15.5 ms. When $\:{g}_{Ks}$ was reduced further to 0.8, evoked responses began to emerge even in the standard sequence, and the difference in response rates between the deviant and control sequences diminished. Finally, at $\:{g}_{Ks}$ = 0.3 (high ACh), evoked responses became weaker across all sequences, and late, scattered responses were more frequently observed. These response patterns were further quantified using post-stimulus time histograms, which measure the average number of spikes per millisecond across trials for each sequence (Fig. 4B).

We then calculated the deviance detection index (DDI), which quantifies the relative response magnitude between the deviant and control sequences (see Methods), in the sample network across different $\:{g}_{Ks}$ values ranging from 1.5 to 0 (Fig. 4C). When ACh was absent ($\:{g}_{Ks}$ = 1.5), the DDI was low (0.17) but not zero, suggesting that the HH neural network, despite lacking any form of plasticity, is still capable of detecting deviant stimuli (see Discussion for further details). Introducing a small amount of ACh ($\:{g}_{Ks}$ = 1.3) increased the DDI to a peak of 0.361. As $\:{g}_{Ks}$ was further reduced, the DDI gradually declined to zero, indicating that the network responses to the deviant and control sequences became increasingly similar (as shown in Fig. 4A).

**Fig. 5:**
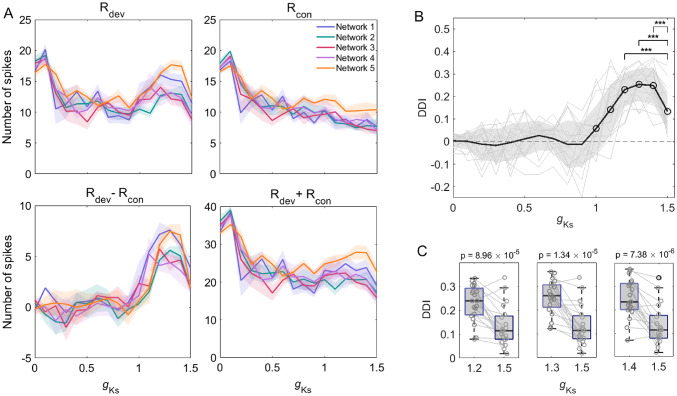
Cholinergic modulation of deviance detection. **A** Average network responses to stimulus A in the A-as-dev ($\:{R}_{dev}$) and A-as-con ($\:{R}_{con}$) sequences. The difference ($\:{R}_{dev}-{R}_{con}$) and sum ($\:{R}_{dev}+{R}_{con}$) are also shown. The DDI is defined as the ratio of the difference to the sum. **B** DDI values plotted against $\:{g}_{Ks}$ across 20 simulations (5 network configurations × 4 stimulation sites). The black line indicates the average trend. DDIs significantly greater than zero (α = 0.05, one-tailed Wilcoxon test with Benjamini–Hochberg method) are marked with black circles. Asterisks indicate values significantly greater than the no-ACh condition ($\:{g}_{Ks}$ = 1.5). **C** Conditions where DDI values ($\:{g}_{Ks}$ = 1.2, 1.3, and 1.4) were significantly higher than at $\:{g}_{Ks}$ = 1.5. Corresponding p-values are shown above each comparison

To ensure that the results were not dependent on the specific choice of stimulus sites or network structures, we repeated the simulation using four different stimulation sites across five different networks. This yielded a total of 20 simulation sets, each with $\:{g}_{Ks}$ values ranging from 0 to 1.5. The network responses for the A-as-dev and A-in-con sequences are shown in Fig. 5A, and the corresponding 20 DDI vs. $\:{g}_{Ks}$ curves are presented in Fig. 5B. To further assess the cholinergic effect on DDI, we compared DDI values across different $\:{g}_{Ks}$ levels to those at $\:{g}_{Ks}$ = 1.5 (no ACh) (pairwise one-tailed t-tests). The results showed that DDI was significantly higher at $\:{g}_{Ks}$ values of 1.4, 1.3, and 1.2 (Fig. 5B). These comparisons are further illustrated in Fig. 5C. These results confirm that a small amount of ACh maximizes deviance detection.

### Robustness of the DDI-$\:{\boldsymbol{g}}_{\boldsymbol{K}\boldsymbol{s}}$ relationship under neuronal and synaptic heterogeneity

To determine whether the relationship between ACh level and DDI (as shown in Fig. 5B) depends on the uniform parameter set used in the main simulations, we repeated the $\:{g}_{Ks}$ sweep in networks with neuronal and synaptic heterogeneity. For each neuron and synapse, parameters were independently sampled from biologically plausible ranges (Table 1), resulting in a unique set of neuronal and synaptic properties for each network realization. Five new networks were generated for each of the five network topologies used in the previous simulations, yielding a total of 25 independent network configurations. The $\:{g}_{Ks}$ sweep (0–1.5 mS/cm²) was then performed on each new configuration.

**Table 1:** Neuronal and synaptic parameters used in the HH network model

Table: Symbol	Parameter name	Value	Literature source	Sensitivity range	Source of range
Membrane properties					
*C*	Membrane capacitance	1 µF/cm²	Fink et al. (2011); Mainen & Sejnowski (1996);	Fixed	–
$\:{g}_{Na}$	Na⁺ maximal conductance	24.0 mS/cm²	Fink et al. (2011); Mainen & Sejnowski (1996);	13.2–24.6 mS/cm²	Stiefel et al. (2009); Prinz et al. (2004);
$\:{g}_{Kdr}$	Delayed-rectifier K⁺ conductance	3.0 mS/cm²	Fink et al. (2011); Mainen & Sejnowski (1996);	2–20 mS/cm²	Pospischil et al. (2008); Borkowski (2009);
$\:{g}_{Ks}$	Slow K⁺ (M-current) conductance	0–1.5 mS/cm²	Fink et al. (2011); Mainen & Sejnowski (1996);	0–1.5 mS/cm²	Roach et al. (2019); Fink et al. (2011);
$\:{g}_{L}$	Leak conductance	0.02 mS/cm²	Fink et al. (2011); Mainen & Sejnowski (1996);	0.016–0.022 mS/cm²	Stiefel et al. (2009); Borkowski (2009);
Reversal potentials					
$\:{E}_{Na}$	Na⁺ reversal potential	55.0 mV	Hodgkin and Huxley (1952); Fink et al. (2011);	50–60 mV	Hille et al. (2001);
$\:{E}_{K}$	K⁺ reversal potential	−90.0 mV	Hodgkin and Huxley (1952); Fink et al. (2011);	−80 mV to − 90 mV	Jalonen et al. (1989)
$\:{E}_{L}$	Leak reversal potential	−60.0 mV	Roach et al. (2019); Fink et al. (2011);	−56.0 mV to − 64.0 mV	Stiefel et al. (2009)
Gate kinetics					
$\:{\mathrm{V}}_{h,Na}$	Na⁺ inactivation half-voltage	−53.0 mV	Roach et al. (2019); Fink et al. (2011);	Fixed	–
$\:{\mathrm{V}}_{h,z}$	M-current activation half-voltage	−39.0 mV	Roach et al. (2019); Fink et al. (2011);	Fixed	–
Synaptic parameters					
$\:{E}_{e}$	Excitatory reversal potential	0 mV	Destexhe et al. (1994); Gabbiani et al. (1994);	Fixed	–
$\:{E}_{i}$	Inhibitory reversal potential	−100 mV	Destexhe et al. (1994); Gabbiani et al. (1994);	Fixed	–
$\tau _{e}$	Excitatory synaptic time constant	2 ms	Destexhe et al. (1994); Abbott et al. (1997);	1.5–3 ms	Destexhe et al., (1994); Häusser & Roth, (1997)
$\tau _{e}$	Inhibitory synaptic time constant	4 ms	Destexhe et al. (1994); Abbott et al. (1997);	4–7.2 ms	Otis & Mody (1992);
*w*	Synaptic weight	1 nS	Destexhe et al. (1994); Abbott et al. (1997);	0.5–4.0 nS	Destexhe et al., (1994); Häusser & Roth, (1997)
*U*	Synaptic utilization factor	0.4	Destexhe et al. (1994); Tsodyks & Markram (1997)	0.1–0.95	Tsodyks & Markram (1997)

Parameters designated as Fixed were held constant at the specified baseline value across all 200 neurons. All other parameters were independently sampled from a uniform distribution within the indicated physiological range. The cited literature supports the baseline values, while the final column lists the sources used to define the corresponding physiological ranges

**Fig. 6:**
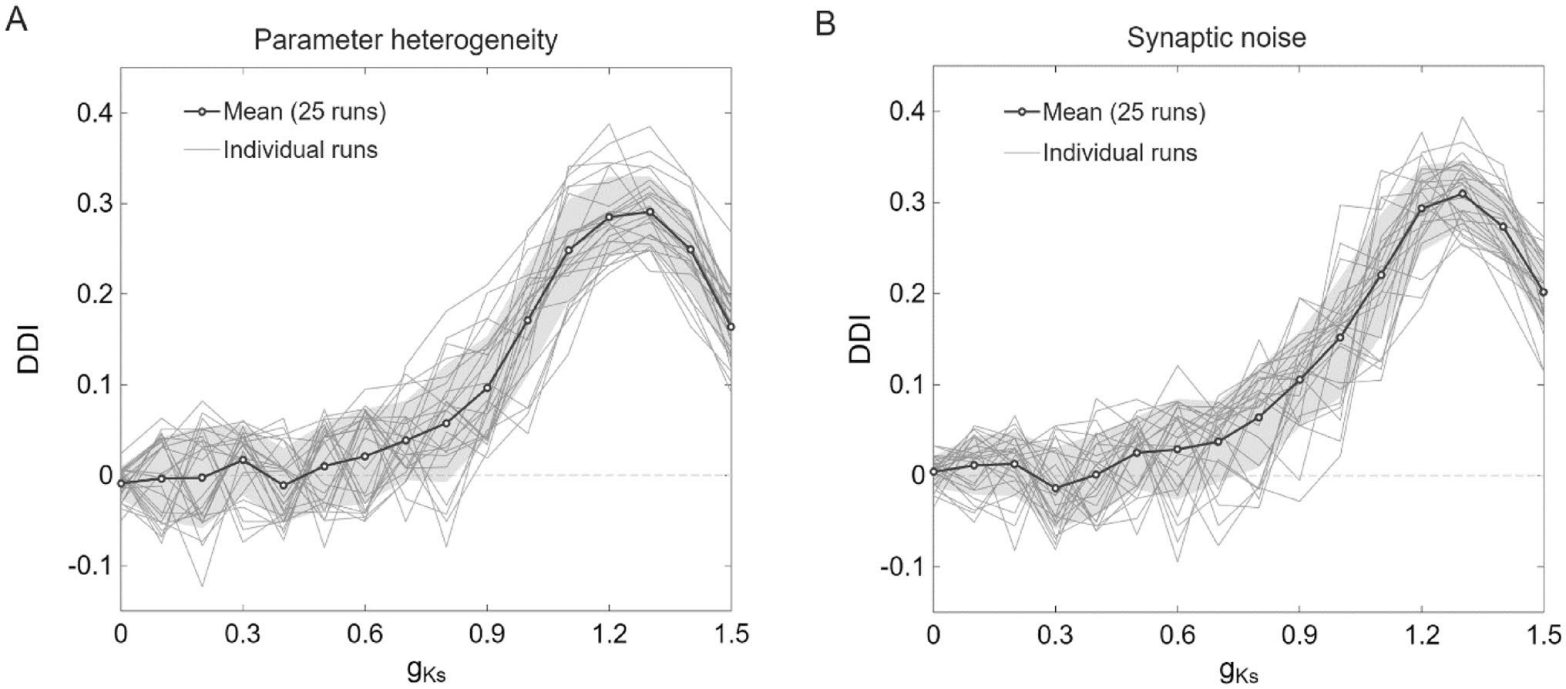
Robustness of the DDI–$\:{g}_{Ks}$ relationship. **A** Robustness under neuronal parameter heterogeneity. The deviance detection index (DDI) is plotted as a function of $\:{g}_{Ks}$ values across five independent network realizations. Each solid gray line represents an individual network realization (25 independent runs in total). The black line with open circles indicates the mean DDI across all 25 runs, and the gray shaded area represents the standard deviation. **B** Robustness under background synaptic noise. The same representation is used as in panel **A**

Across all configurations, the relationship between $\:{g}_{Ks}$ and DDI was preserved (Fig. 6A). DDI values remained near zero at low $\:{g}_{Ks}$, increased progressively at intermediate levels, peaked consistently around $\:{g}_{Ks}\approx\:1.2-1.3$ mS/cm², and declined at higher $\:{g}_{Ks}$ values. These results indicate that the relationship between ACh level and genuine DD is robust to biologically realistic neuronal heterogeneity and does not depend on the assumption of a homogeneous network.

### Robustness of the DDI-$\:{\boldsymbol{g}}_{\boldsymbol{K}\boldsymbol{s}}$ relationship under background noise

To assess whether background spontaneous activity influences the relationship between ACh level and DDI, we introduced Gaussian white noise into the membrane current of each neuron at every integration step (noise amplitude $\sigma = 1\mu {\mathrm{A}}/{\mathrm{cm}}^{2}$; see details in Methods). The $\:{g}_{Ks}$ sweep (0–1.5 mS/cm²) was then repeated on the five original networks (without parameter heterogeneity), with five independent noise realizations for each network, yielding 25 additional simulations. Across all realizations, the relationship between $\:{g}_{Ks}$ and DDI was preserved (Fig. 6B), indicating that genuine DD remains robust across biologically plausible levels of background synaptic noise.

### Cholinergic modulation of local suppression via spike frequency adaptation

In this section, we aim to provide a mechanistic explanation of the observed cholinergic facilitation of DD. We first validated an existing theory of genuine DD proposed by Kern et al. (Kern and Chao 2023), which suggests that either neuronal threshold adaptation or synaptic depression—two forms of short-term plasticity—reduces the propagation of evoked responses to standard stimuli (in this case, stimulation B). This suppression frees up neural resources for responding to deviant stimuli (stimulation A). Such local suppression is less prominent in the control sequence, where stimuli are spatially distributed, which together contributes to a higher DDI. Although our model does not include explicit rules for neuronal threshold adaptation or synaptic depression, SFA can be induced by ACh in HH neurons (Cui and Strowbridge 2019; Roach et al. 2019).

**Fig. 7:**
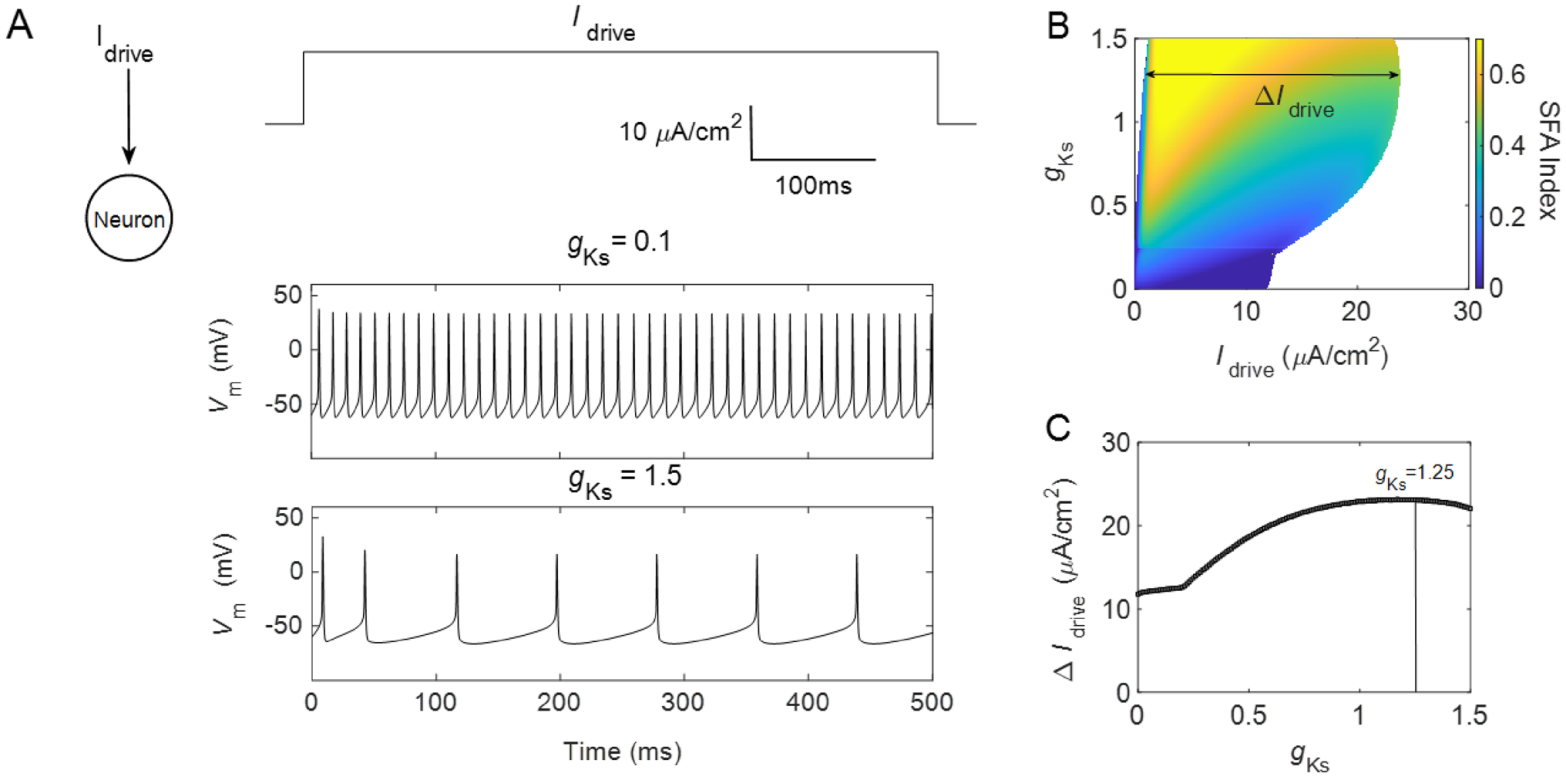
Cholinergic modulation of spike frequency adaptation. **A** Responses of a HH neuron to sustained current stimulation under different $\:{g}_{Ks}$ values. **B** SFA as a function of $\:{g}_{Ks}$ and stimulation current ($\:\varDelta\:{I}_{drive}$). White areas indicate conditions where SFA is absent (see main text for details). For each $\:{g}_{Ks}$ level, $\:\varDelta\:{I}_{drive}$ is defined as the range of current over which SFA occurs. **C** $\:\varDelta\:{I}_{drive}$ as a function of $\:{g}_{Ks}$. The maximum $\:\varDelta\:{I}_{drive}$ is observed at $\:{g}_{Ks}$ = 1.25 (indicated by the vertical line)

Figure 7A illustrates SFA in response to a 500-ms current pulse. At $\:{g}_{Ks}$= 0.1, spiking activity was sustained with little change in inter-spike intervals (ISIs). In contrast, at $\:{g}_{Ks}$= 1.5, firing rates were lower, and ISIs progressively lengthened, indicating SFA. This adaptation was quantified by the change in ISI over time (see Methods). As shown in Fig. 7B, SFA depends on both input current and $\:{g}_{Ks}$. In general, stronger input currents produced weaker SFA, whereas higher $\:{g}_{Ks}$ values produced stronger SFA. Under very strong stimulation, however, SFA disappeared (white regions in Fig. 7B). In these cases, neurons fired only a few spikes before becoming trapped in a highly depolarized state (e.g., − 20 mV), resulting in sodium channel inactivation and cessation of spiking (Supplementary Figure S1).

To quantify the propensity of a network to express SFA, we defined $\:\varDelta\:{I}_{drive}\:$as the range of input currents over which SFA occurs (Fig. 7B). Unlike the magnitude of SFA at a particular current level, $\:\varDelta\:{I}_{drive}\:$reflects the width of the SFA-producing regime. Consequently, larger $\:\varDelta\:{I}_{drive}\:$values increase the likelihood that fluctuating synaptic inputs will engage SFA in postsynaptic neurons. When $\:\varDelta\:{I}_{drive}\:$was plotted against $\:{g}_{Ks}$ (Fig. 7C), it reached a maximum value of 23 at $\:{g}_{Ks}$ = 1.25, close to the condition that produced the highest DDI. This correspondence supports the idea that ACh modulates DDI through SFA, a local suppression mechanism that limits the propagation of activity evoked by repeated standard stimuli.

### Cholinergic modulation of global synchronization via phase locking

**Fig. 8:**
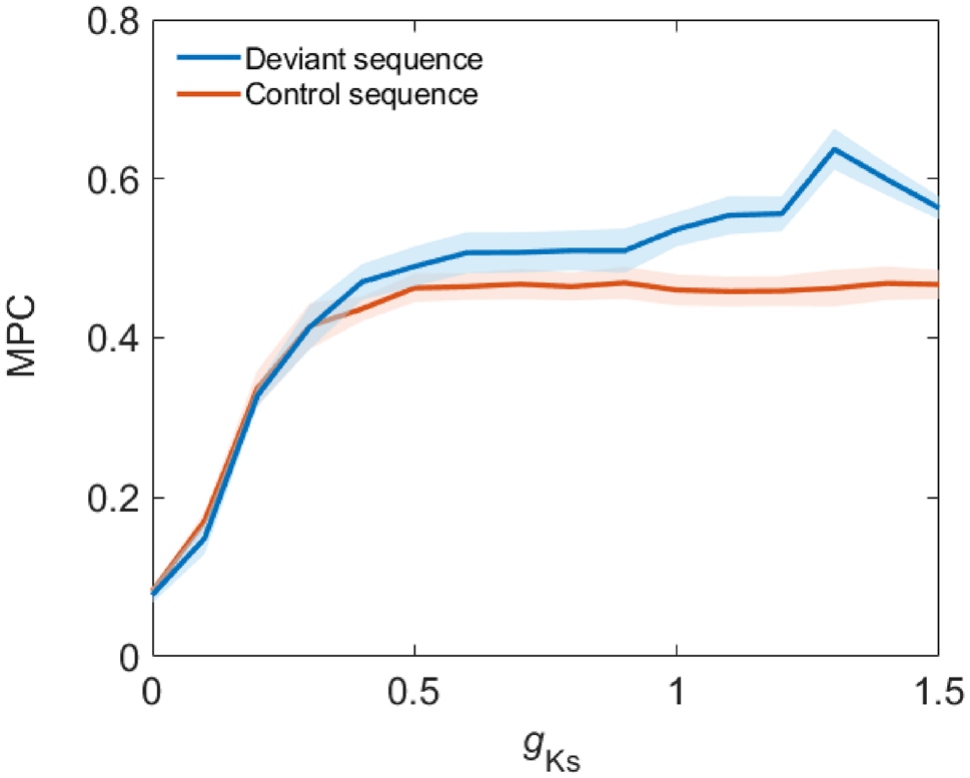
Cholinergic modulation of phase-locking in the network. Mean phase coherence (MPC), a measure of average synchrony between pairs of neurons, was computed for the A-as-dev and A-in-con sequences under different $\:{g}_{Ks}$ values. MPC was calculated across all neuron pairs and 5 simulations (5 network configurations). The mean and standard deviation are shown

In addition to local suppression, cholinergic modulation can also promote phase locking, where neuronal firing becomes more synchronized (Roach et al. 2019). We quantified phase locking using mean phase coherence (MPC), which reflects the average synchrony between pairs of neurons, ranging from 0 (random firing) to 1 (perfect phase locking). Figure 8 shows MPC values across different $\:{g}_{Ks}$ levels for all five networks under both deviant and control sequences. When $\:{g}_{Ks}$ > 0.5, MPC for the deviant sequence becomes greater than that for the control sequence, reaching a maximum at $\:{g}_{Ks}$ = 1.3, where MPC peaks at 0.64. This result suggests that the network’s ability to synchronize is maximized at $\:{g}_{Ks}$ = 1.3 under the deviant sequence. At this point, the probability of neurons firing simultaneously increases, enhancing the response to deviant stimuli. This, in turn, amplifies the difference between responses to deviant and control sequences, leading to a maximal DDI.

**Fig. 9:**
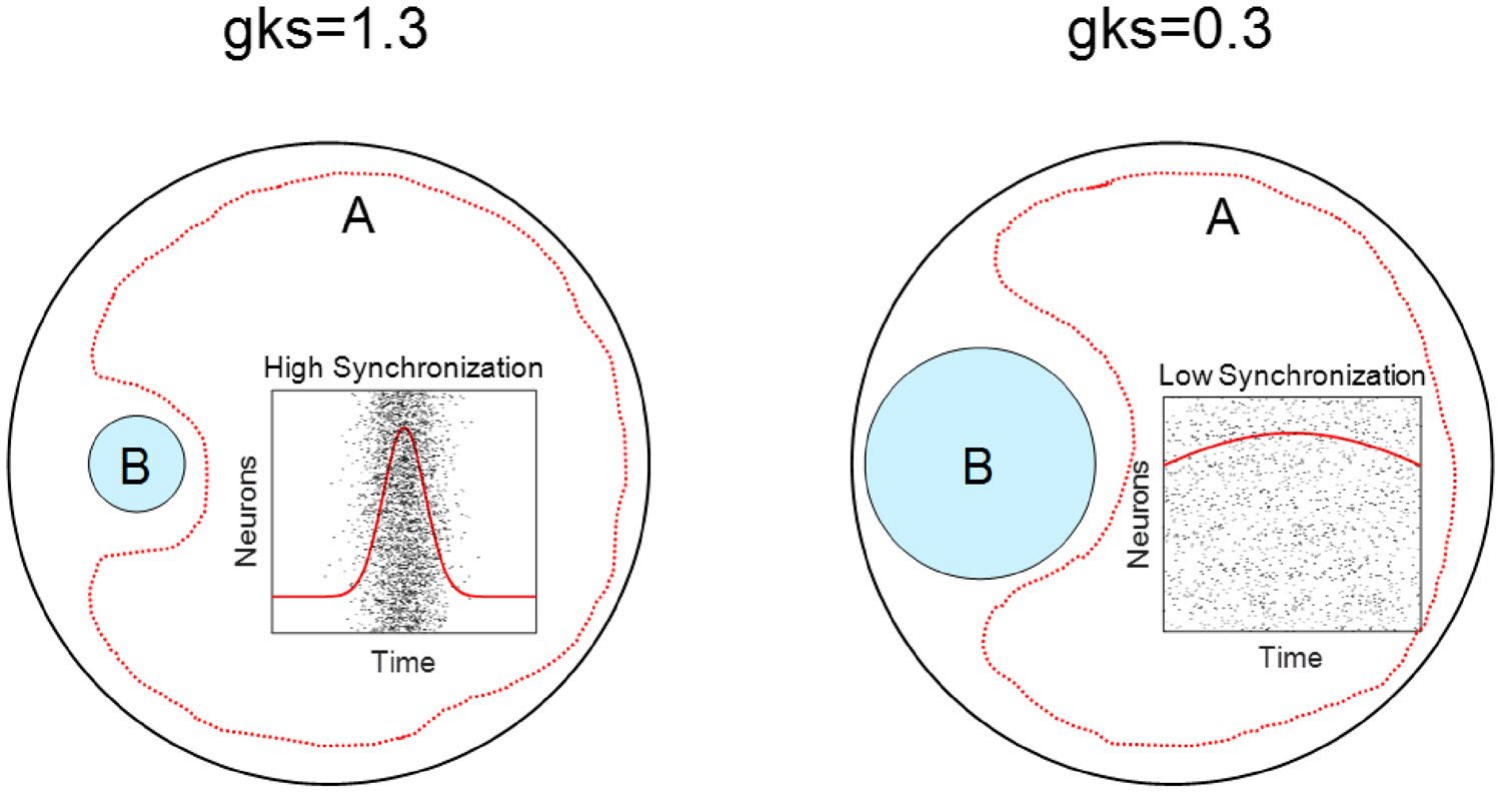
Conceptual model of cholinergic modulation in genuine DD. In the A-as-dev sequence, when $\:{g}_{Ks}$ = 1.3 (left), spike frequency adaptation is strongest. As a result, repeated standard stimulation (stimulus B) suppresses activity near the electrode, preventing further propagation of evoked responses (indicated by the small blue circle). This suppression frees up the rest of the network (red dotted area) to respond more robustly to the deviant stimulus A. In addition, phase-locking is maximal at $\:{g}_{Ks}$ around 1.3, resulting in stronger synchronization and a more prominent network-wide response to A. In contrast, when $\:{g}_{Ks}$ deviates from 1.3 (e.g., 0.3; right panel), both local suppression and global synchronization are reduced, leading to a weaker deviant response

Together, we propose that at $\:{g}_{Ks}$ = 1.3, the combination of maximal local suppression, driven by the strongest SFA to the standard stimulus B, which frees the greatest amount of neural resources for the deviant stimulus A in the A-as-dev sequence, and the strongest globally synchronized response to A due to maximal phase locking, results in the most robust evoked response to A. This interplay results in the maximal DDI (see illustration in Fig. 9).

### Dynamical explanation of the DDI–$\:{\boldsymbol{g}}_{\boldsymbol{K}\boldsymbol{s}}$ relationship

When $\:{g}_{Ks}<0.5$, suppression of the M-current produces Type 1 excitability. Under this regime, SFA is largely absent (Fig. 7C), preventing repeated standard stimuli from suppressing local activity. At the same time, the monophasic phase-response curve does not support selective phase locking, resulting in similar MPC values for the deviant and control sequences (Fig. 8). Consequently, both local suppression and global synchronization are weak, producing DDI values close to zero.

As $\:{g}_{Ks}$increases to 1.0–1.3, neurons transition to Type 2 excitability. SFA becomes strongest (Fig. 7C), maximizing suppression of repeated standard stimuli and freeing network resources for deviant responses. Simultaneously, the biphasic phase-response curve supports selective synchronization, producing the largest MPC difference between the deviant and control conditions (Fig. 8). The coincidence of maximal SFA and maximal synchronization results in the peak DDI near $\:{g}_{Ks}=1.3$.

At $\:{g}_{Ks}=1.5$, neurons typically fire only a single spike in response to each stimulus. Although the M-current remains strong, sparse spiking limits both adaptation and network synchronization, reducing the difference between deviant and control responses. Consequently, DDI decreases from its optimum.

### Short-term synaptic depression further enhances deviance detection

**Fig. 10:**
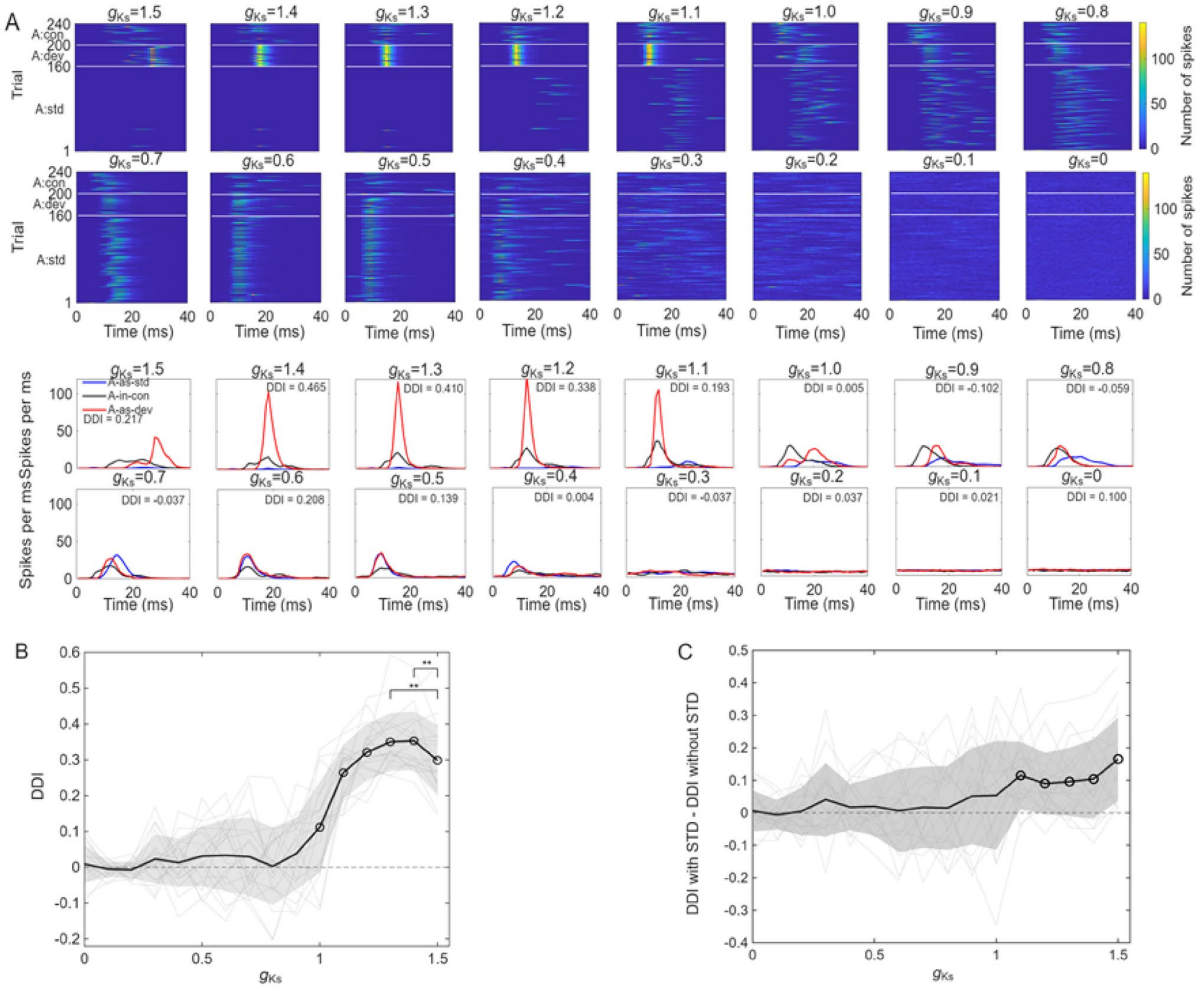
Short-term synaptic depression enhances deviance detection. **A** DD in a sample network with STD. Network responses to the target stimulus A at different $\:{g}_{Ks}$.values are shown on the top two rows, and the corresponding post-stimulus time histograms are shown in the bottom two rows. **B** Cholinergic modulation of DD with STD. Each gray line represents the DDI as a function of $\:{g}_{Ks}$ for one of the 20 simulations (5 network configurations × 4 stimulation sites), and the black line represents the mean across all simulations. Asterisks indicate DDI values that are significantly greater than the baseline at $\:{g}_{Ks}$ =1.5 (paired t-test, *p* < 0.01). Open circles on the mean curve indicate $\:{g}_{Ks}$ values for which the mean DDI is significantly greater than zero. **C** Comparison of DD with and without short-term synaptic depression. The difference in DDI (DDI with STD minus DDI without STD) is plotted across 20 simulations. Open circles indicate values that are significantly greater than zero

To examine the contribution of short-term synaptic depression (STD) to DD, we incorporated STD into excitatory synapses while maintaining the same network architecture and stimulation protocol. The behavior of a representative network is illustrated in Fig. 10A, which shows spike raster plots and post-stimulus time histograms across a range of $\:{g}_{Ks}$ values.

Across all tested $\:{g}_{Ks}\:$values, the addition of STD increased DDI relative to models with cholinergic modulation alone. This enhancement was most pronounced near $\:{g}_{Ks}\:$= 1.3, where DDI increased from 0.316 to 0.410. To assess the robustness of this effect, we plotted DDI as a function of $\:{g}_{Ks}\:$across 20 independent simulations (Fig. 10B). The relationship between $\:{g}_{Ks}\:$and DDI was preserved, with peak DDI values occurring near $\:{g}_{Ks}\:$= 1.3–1.4. Direct comparison of models with and without STD confirmed that STD significantly enhanced DDI across a broad parameter range ($\:{g}_{Ks}$= 1.1–1.5; Fig. 10C).

These findings indicate that STD and cholinergic modulation act synergistically to promote genuine DD. Whereas cholinergic modulation regulates neuronal excitability and network synchronization, STD provides an additional adaptive mechanism that selectively suppresses responses to repeated standard stimuli, thereby increasing the contrast between deviant and control responses.

## Discussion

We use a HH neural network to show that cholinergic modulation can suppress neuronal firing and enhance interneuronal synchronization, enabling genuine DD. This finding indicates that spatially distributed neural computation allows the network to retain a memory of both the stimulus identity and its regularity, and suggests that genuine DD can arise through purely feedforward signaling, without the need for top-down modulation and long-term plasticity.

### Origins of genuine DD

Our results show that even when $\:{g}_{Ks}$ is set to 1.5, representing a low ACh state, DDI is 0.17, indicating a residual ability to detect deviations (see Fig. 3). This effect can be attributed to SFA, which remains active at $\:{g}_{Ks}$ = 1.5 (Cui and Strowbridge 2019; Roach et al. 2019). SFA is a non-synaptic plasticity mechanism that reduces neuronal firing rates over repeated stimulation. It operates similarly to threshold adaptation (Fontaine et al. 2014; Shaban et al. 2021), but while threshold adaptation raises the spiking threshold, SFA reduces spike counts by lowering discharge frequency. Kern & Chao (Kern and Chao 2023) demonstrated that threshold adaptation alone is sufficient for DD. They proposed a dual mechanism of local fatigue and global recovery: neurons near the repeated standard stimulus become suppressed (local fatigue), which prevents widespread activation. As a result, the rest of the network remains responsive (global recovery), allowing strong responses to unexpected stimuli. Our cholinergic HH model replicates this mechanism: ACh-regulated SFA induces local fatigue around repetitive stimuli, preserving broader network sensitivity for detecting deviants.

Although both SFA and network synchronization were associated with enhanced DD, the present model does not establish them as independent mechanisms. Both effects emerge from modulation of the same biophysical parameter, the M-current conductance ($\:{g}_{Ks}$), and may therefore represent different manifestations of a common cholinergic process rather than distinct causal pathways. Consequently, the framework summarized in Fig. 9 should be viewed as a conceptual interpretation of the model rather than evidence for two dissociable mechanisms. Future studies that independently manipulate neuronal adaptation and synchronization, for example through heterogeneous cholinergic modulation or targeted circuit perturbations, will be required to determine their respective contributions to deviance detection.

Kern and Chao (2023) reported a synergistic effect between threshold adaptation (functionally equivalent to SFA in our model) and short-term synaptic depression. Both neuronal and synaptic forms of short-term plasticity can support genuine DD, and their effects can be additive. Consistent with this prediction, incorporating short-term synaptic depression into our model further enhanced DD (Fig. 10). Whereas cholinergic modulation regulates neuronal excitability and network synchronization, short-term synaptic depression provides an additional adaptive mechanism that selectively suppresses responses to repeated standard stimuli, thereby increasing the contrast between deviant and control responses. We therefore propose that any short-term adaptive mechanism operating across space can encode information about both stimulus identity and its regularity, thereby supporting genuine DD. This framework does not preclude a role for long-term plasticity in genuine DD (Azouz and Gray 2003; Bounds and Adesnik 2025; Derveer et al. 2023; Obara et al. 2023; Parras et al. 2017).

### Synchronization and latency shifts: temporal dynamics of deviance detection

We found that network synchronization was highest at $\:{g}_{Ks}$ = 1.3 in the deviant sequence (Fig. 8), which corresponds to a state of low ACh and Type 2 neuronal excitability. According to the mechanisms described by Roach et al. (Roach et al. 2019), Type 2 neurons possess two key properties that facilitate synchronization: (1) a flat frequency-current (f/I) curve (see f/I curves of our model under different $\:{g}_{Ks}$ values in Supplementary Figure S2), which ensures firing rates are uniform across the network, and (2) a biphasic phase-response curve (see phase-response curves of our model under different $\:{g}_{Ks}$ values in Supplementary Figure S3), which allows neurons to both advance and delay their spike times to align with each other. At $\:{g}_{Ks}$ = 1.3, these properties yield a latency of 15.5 ms, not the latency of single neurons, but the timing of a collective, synchronous burst characteristic of a phase-coding regime. When $\:{g}_{Ks}$ decreased to 1.0 (see Supplementary Figure S4), latency advanced to 11.5 ms, consistent with a shift to rate coding (Type 1 excitability). In this high-ACh state, the neuronal f/I curve becomes steeper, producing high gain: small inputs trigger a large, rapid increase in firing rate. The network then shifts from encoding information in the precise timing of spikes within a synchronized population to encoding it in the firing rate of individual neurons. Thus, $\:{g}_{Ks}$ functions as a dynamic control parameter, switching the network between two computational regimes: phase coding with synchronized bursts versus rate coding with rapid, individual responses, and in doing so, modulates the temporal properties of DD.

### Biological relevance of $\:{\boldsymbol{g}}_{\boldsymbol{K}\boldsymbol{s}}$ and cholinergic modulation

We showed that moderate ACh levels enhance DD (Figs. 3 and 4). In our model, cholinergic modulation of neuronal excitability is implemented by varying the maximal conductance of the M-current (a slow, muscarinic receptor-regulated potassium current) represented by the parameter $\:{g}_{Ks}$. This approach is grounded in well-established findings that ACh suppresses the M-current, thereby increasing neuronal excitability (Brown and Adams 1980; McCormick and Prince 1985). High ACh levels, which strongly block the M-current, are modeled as a low conductance value ($\:{g}_{Ks}\:$= 0), producing Type 1 excitability. Conversely, low ACh levels, where the M-current remains active, are modeled as a high conductance ($\:{g}_{Ks}\:$= 1.5), producing Type 2 excitability (Roach et al. 2019). This parameterization is quantitatively supported by the concentration-response relationship reported by Adams et al. (1982), who found that the ACh agonist muscarine inhibits the M-current in a dose-dependent manner, with near-maximal suppression (~ 80%) at ≥ 10 µM and a half-maximal effective concentration (EC₅₀) of ~ 3 µM. Thus, in our model, the $\:{g}_{Ks}\:$= 0 condition could correspond directly to the experimentally observed state of near-complete M-current blockade under high cholinergic tone (≥ 10 µM muscarine).

The finding that deviance detection is maximized at an intermediate $\:{g}_{Ks}$value is not merely a model-dependent result, but is supported by the known dose-response relationship between acetylcholine and M-current suppression. Experimental measurements show that muscarinic suppression of the M-current follows a Hill function with EC₅₀ $\:\approx\:0.97$ µM, Hill coefficient *\:n=1.3*, and maximum suppression $\:{R}_{\mathrm{m}\mathrm{a}\mathrm{x}}=0.78\:$(Hoshi et al. 2005). Assuming an unmodulated conductance of $\:{g}_{Ks}=1.5$, the optimal value of $\:{g}_{Ks}=1.3\:$corresponds to approximately 86.7% residual M-current conductance, which, based on the experimentally derived Hill equation, is achieved at an estimated ACh concentration of ~ 0.29 µM. This concentration falls within the physiological range associated with low-to-moderate cholinergic tone during quiet waking (Hasselmo and McGaughy 2004). Nevertheless, a precise conversion between $\:{g}_{Ks}\:$and absolute ACh concentration will require cell type-specific electrophysiological calibration. Future experiments will test whether this predicted concentration similarly enhances genuine DD in neuronal cultures.

### Modeling limitations regarding cholinergic signaling

One limitation of the present model is that cholinergic modulation is represented as a static change in M-current conductance rather than by explicitly modeling acetylcholine release, diffusion, receptor activation, or intracellular signaling. Because M1 muscarinic receptor signaling operates on relatively slow timescales compared with membrane potential dynamics, the model should be interpreted as describing neuronal and network activity after cholinergic modulation has reached a quasi-steady state. Consequently, the synchronization and deviance detection effects reported here reflect the computational consequences of different cholinergic states rather than the temporal dynamics of cholinergic signaling itself. Future studies incorporating dynamic acetylcholine release and receptor kinetics will be necessary to address these processes.

A second limitation concerns network architecture. The simulated network (200 neurons distributed within a 4 mm radius; ~398 cells/cm²) is substantially less dense than dissociated cortical or hippocampal cultures, which typically contain several thousand neurons per cm². The spatial organization in our model was intended primarily to impose distance-dependent connectivity rather than to reproduce the physical density of biological cultures. Future work should examine whether the cholinergic effects reported here generalize to larger and more densely connected networks.

### Spatial heterogeneity of ACh and implications for modeling

Our current model assumes a homogeneous distribution of ACh by using the same $\:{g}_{Ks}$ value across all neurons in the network. While earlier models similarly treated ACh modulation as spatially diffuse (Li et al. 2018; Picciotto et al. 2012), more recent studies highlight its spatially localized and dynamic nature (Lohani et al. 2022; Zaborszky et al. 2015). Anatomical and functional evidence, such as spatially restricted cholinergic release (Disney and Higley 2020; Parikh and Sarter 2006), suggests that ACh can modulate specific cortical subnetworks. Yang et al. (Yang et al. 2023) demonstrated that such spatial heterogeneity can drive synaptic reorganization and feature binding. In particular, regions with higher ACh concentrations (reflected as lower $\:{g}_{Ks}$ values) show enhanced excitability and plasticity, leading to localized network strengthening. Lohani et al. (Lohani et al. 2022) further showed that spatially distinct ACh signaling can dynamically regulate cortical states in behaviorally relevant ways. In future work, we aim to incorporate spatial heterogeneity of ACh into our model to better understand its role in DD.

### Comparison with other ACh models

Traditional ACh models used static modulation of HH-type conductance. More recent work, such as Codianni & Rubin (Codianni and Rubin 2023), introduced dynamic models that simulate extracellular ACh concentration using differential equations, incorporating spike-triggered release, degradation, and receptor kinetics. Their model affects both M-current and synaptic input in real time, enabling feedback-based modulation of network states. Fang et al. constructed a cholinergic-regulated Hopfield network and investigated how the local and global inhibitory balance and spike frequency adaptation of acetylcholine regulation promote flexible transitions between different memory states (Fang et al. 2026). Sajedin et al. (Sajedin et al. 2019) implemented a leaky integrate-and-fire (LIF) network to simulate cholinergic effects on orientation selectivity in visual cortex. Their model used $\:{g}_{Ks}$ modulation to emulate ACh’s role in sharpening tuning curves and reducing response variability. Our model, while simpler, is consistent with this framework: $\:{g}_{Ks}$ regulates ACh-like effects on excitability, synchronization, and deviance detection.

## Methods

### Neuronal model

The model includes a fast, inward Na^+^ current, a delayed rectifier K^+^ current, a leakage current, and a slow, low-threshold M-type K+ current. With membrane capacitance *C* = 1 $\:\mu\:F/{cm}^{2}$, voltage $\:{V}_{i}$ in millivolts, and time *t* in milliseconds, the current balance equation for the *i*-th cell is:

1 $$ \begin{aligned} C\frac{{dV_{i} }}{{dt}} = & - {\mathrm{g}}_{{{\mathrm{Na}}}} {\mathrm{m}}_{{\infty \:}}^{3} h\left( {{\mathrm{V}}_{{\mathrm{i}}} - {\mathrm{E}}_{{{\mathrm{Na}}}} } \right) - {\mathrm{g}}_{{K_{{dr}} }} n^{4} \left( {{\mathrm{V}}_{{\mathrm{i}}} - {\mathrm{E}}_{{\mathrm{K}}} } \right) \\ & \quad - g_{{Ks}} z\left( {{\mathrm{V}}_{{\mathrm{i}}} - {\mathrm{E}}_{{\mathrm{K}}} } \right) - {\mathrm{g}}_{{\mathrm{L}}} \left( {{\mathrm{V}}_{{\mathrm{i}}} - {\mathrm{E}}_{{\mathrm{L}}} } \right) + {\mathrm{I}}_{{{\mathrm{drive}}}}^{i} + {\mathrm{I}}_{{{\mathrm{syn}}}}^{{\mathrm{i}}} \\ \end{aligned} $$

$\:{I}_{drive}$ was a constant externally applied current. For the Na+ channel, activation was instantaneous, with the steady-state function:

2 $$\:{m}_{\infty\:}\left(V\right)=\frac{1}{1+{e}^{(-V-30.0)/9.5}}$$

The inactivation gating variable $\:{h}_{i}$ was described by:

3 $$\:\frac{\mathrm{d}{h}_{i}}{\mathrm{d}\mathrm{t}}=\frac{{\mathrm{h}}_{{\infty\:}}\left({V}_{i}\right)-{h}_{i}}{{{\uptau\:}}_{\mathrm{h}}\left({V}_{i}\right)}$$

$$\:\mathrm{w}\mathrm{h}\mathrm{e}\mathrm{r}\mathrm{e}\:{\mathrm{h}}_{{\infty\:}}\left(\mathrm{V}\right)=\frac{1}{1+{\mathrm{e}}^{(\mathrm{V}+53.0)/7.0}}\:\mathrm{a}\mathrm{n}\mathrm{d}\:{{\uptau\:}}_{\mathrm{h}}\left(\mathrm{V}\right)=0.37+\frac{2.78}{1+{\mathrm{e}}^{(\mathrm{V}+40.5)/6.0}}.$$

The delayed rectifier K^+^ current was gated by $\:{n}_{i}$:

4 $$\:\frac{\mathrm{d}{n}_{i}}{\mathrm{d}\mathrm{t}}=\frac{{\mathrm{n}}_{{\infty\:}}\left({V}_{i}\right)-{n}_{i}}{{{\uptau\:}}_{\mathrm{n}}\left({V}_{i}\right)}$$

$$\:\mathrm{w}\mathrm{h}\mathrm{e}\mathrm{r}\mathrm{e}\:{\mathrm{n}}_{{\infty\:}}\left(\mathrm{V}\right)=\frac{1}{1+{\mathrm{e}}^{(-\mathrm{V}-30.0)/10.0}}\:\mathrm{a}\mathrm{n}\mathrm{d}\:{{\uptau\:}}_{\mathrm{n}}\left(\mathrm{V}\right)=0.37+\frac{1.85}{1+{\mathrm{e}}^{(\mathrm{V}+27.0)/15.0}}.$$

The slow, low threshold K^+^ current was gated by $\:{z}_{i}$:

5 $$\:\frac{\mathrm{d}{z}_{i}}{\mathrm{d}\mathrm{t}}=\frac{{\mathrm{z}}_{{\infty\:}}\left({V}_{i}\right)-{z}_{i}}{75}$$

where $$\:{\mathrm{z}}_{{\infty\:}}\left(\mathrm{V}\right)=\frac{1}{1+{\mathrm{e}}^{(-\mathrm{V}-39.0)/5.0}}$$.

Other parameters were: $\:{g}_{Na}$ = 24.0 $\:mS/{cm}^{2}$, $\:{g}_{Kdr}$ = 3.0 $\:mS/{cm}^{2}$, $\:{g}_{L}$ = 0.02 $\:mS/{cm}^{2}$, $\:{E}_{Na}$ = 55.0 mV, $\:{E}_{K}$ = −90.0 mV, and $\:{E}_{L}$ = −60.0 mV. The maximal conductance of the slow K+ current $\:{g}_{Ks}^{i}$ varied between 1.5 mS/cm² (no ACh) and 0 mS/cm² (strong ACh). Synaptic interactions were modeled as transient changes in conductance.

### Synaptic dynamics

Synaptic currents were modeled using a conductance-based approach:

6 $$\:{I}_{syn}={g}_{e}\left({\mathrm{V}}_{\mathrm{i}}-{\mathrm{E}}_{\mathrm{e}}\right)+{g}_{i}\left({\mathrm{V}}_{\mathrm{i}}-{\mathrm{E}}_{\mathrm{i}}\right)$$

Here, $\:{\mathrm{E}}_{\mathrm{e}}$ = 0 mV and $\:{\mathrm{E}}_{\mathrm{i}}$ = −100 mV represent the excitatory and inhibitory reversal potentials, respectively. The synaptic conductances evolved as:

$$\:{{\uptau\:}}_{\mathrm{e}}\frac{\mathrm{d}{g}_{e}}{\mathrm{d}\mathrm{t}}=-{g}_{e}+{\sum\:}_{j\propto\:E}Uw\delta\:(t-\widehat{{t}_{j}})$$

7 $$\:{{\uptau\:}}_{\mathrm{i}}\frac{\mathrm{d}{g}_{i}}{\mathrm{d}\mathrm{t}}=-{g}_{i}+{\sum\:}_{j\propto\:I}w\delta\:\left(t-\widehat{{t}_{j}}\right)$$

The time constants $\:{{\uptau\:}}_{\mathrm{e}}$ = 2 ms and $\:{{\uptau\:}}_{\mathrm{i}}$ = 4 ms emulate AMPA and GABA-A receptor dynamics, respectively. The synaptic weight w was fixed at 1 for all connections. The Dirac delta function δ(.) denotes a spike at time $\:{t}_{j}$. The synaptic utilization factor U = 0.4 modeled the constant release probability at excitatory synapses.

### Short-term depression

STD was implemented for excitatory synapses only:

8 $$\:{{\uptau\:}}_{\mathrm{e}}\frac{\mathrm{d}{g}_{e}}{\mathrm{d}\mathrm{t}}=-{g}_{e}+{\sum\:}_{j\propto\:E}U{x}_{j}w\delta\:(t-{\widehat{t}}_{j})$$

Here, $\:{x}_{j}$ is a presynaptic depression variable defined per neuron:

9 $$\:{{\uptau\:}}_{\mathrm{x}}\frac{\mathrm{d}{x}_{j}}{\mathrm{d}\mathrm{t}}=\left(1-{x}_{j}\right)-U{x}_{j}\delta\:(t-\widehat{t})$$

The recovery time constant was $\:{{\uptau\:}}_{\mathrm{x}}$ = 150 ms, and the release fraction was U = 0.4, consistent with auditory cortex data. This model excluded stochastic release, assuming deterministic transmission behavior.

### Network model

We simulated a cortical microcircuit consisting of 200 HH neurons placed randomly within a 2D circular region (radius = 4.0 mm). Neurons were 80% excitatory and 20% inhibitory. Connectivity followed a distance-dependent probabilistic rule: each neuron projected to 10 random targets within a given radius ($\:{r}_{exc}$ = 2 mm, $\:{r}_{inh}$ = 1 mm for excitatory and inhibitory neurons, respectively). Conduction delays were neglected. Synaptic weights were fixed at 1 nS. Five network variants were generated using different random seeds.

### Parameter heterogeneity

To capture the biological variability characteristic of real cortical circuits, we introduced parameter heterogeneity across the neuronal population in our computational model. Rather than assigning uniform, population-wide values, each neuron was assigned a unique set of biophysical parameters drawn independently from a uniform distribution spanning physiologically plausible bounds. This approach reflects the well-documented variability in ion channel expression levels, membrane composition, and synaptic efficacy observed across cortical neurons in vivo.

The randomization protocol was applied to intrinsic membrane conductances ($\:{\mathrm{g}}_{\mathrm{N}\mathrm{a}}$, $\:{\mathrm{g}}_{{K}_{dr}}$, $\:{\mathrm{g}}_{\mathrm{L}}$), ion channel reversal potentials ($\:{\mathrm{E}}_{\mathrm{N}\mathrm{a}}$, $\:{\mathrm{E}}_{\mathrm{K}}$, $\:{\mathrm{E}}_{\mathrm{L}}$), and synapse-specific properties including excitatory and inhibitory decay time constants ($\:{{\uptau\:}}_{\mathrm{e}}$, $\:{{\uptau\:}}_{\mathrm{i}}$), synaptic weights w, and utilization factors *\:U*. To prevent the generation of non-physiological values, the distribution boundaries were strictly constrained within empirically derived ranges sourced from the published literature. The baseline values, sensitivity ranges, and the corresponding primary literature sources are detailed in Table 1. The slow K+ (M-current) conductance $\:{g}_{Ks}$ was held constant across all neurons throughout these analyses, serving as the principal control variable for the deviance detection investigations. We generated five distinct network topologies. For each of these five networks, the heterogeneous physiological parameters were randomly sampled five times, producing 25 independent simulation configurations. By systematically running the $\:{g}_{Ks}$ parameter sweep across these configurations.

### Stochastic background noise

To verify the robustness of the simulation results to spontaneous background neural activity, Gaussian white noise current injection was incorporated into the network model. At each integration time step, an independent noise current drawn from a zero-mean Gaussian distribution was added directly to the membrane voltage equation of every neuron:

10 $$\:{\mathrm{I}}_{\mathrm{n}\mathrm{o}\mathrm{i}\mathrm{s}\mathrm{e}}^{\mathrm{i}}\left(\boldsymbol{t}\right)=\sigma\:\xi\:\left(t\right)$$

where $\sigma = 1\mu {\mathrm{A}}/{\mathrm{cm}}^{2}$ is the noise amplitude (standard deviation), and $\:\xi\:\left(t\right)$ are independent and identically distributed standard normal variates drawn freshly at every time step for every neuron *i*. This noise term was incorporated additively into the membrane voltage equation:

11 $$ \begin{aligned} \:C\frac{{dV_{i} }}{{dt}} = & - {\mathrm{g}}_{{{\mathrm{Na}}}} {\mathrm{m}}_{{\infty \:}}^{3} h\left( {{\mathrm{V}}_{{\mathrm{i}}} - {\mathrm{E}}_{{{\mathrm{Na}}}} } \right) - {\mathrm{g}}_{{K_{{dr}} }} n^{4} \left( {{\mathrm{V}}_{{\mathrm{i}}} - {\mathrm{E}}_{{\mathrm{K}}} } \right) \\ & \quad - g_{{Ks}} z\left( {{\mathrm{V}}_{{\mathrm{i}}} - {\mathrm{E}}_{{\mathrm{K}}} } \right) - {\mathrm{g}}_{{\mathrm{L}}} \left( {{\mathrm{V}}_{{\mathrm{i}}} - {\mathrm{E}}_{{\mathrm{L}}} } \right) + {\mathrm{I}}_{{{\mathrm{drive}}}}^{i} + {\mathrm{I}}_{{{\mathrm{syn}}}}^{{\mathrm{i}}} + {\mathrm{I}}_{{{\mathrm{noise}}}}^{{\mathrm{i}}} \\ \end{aligned} $$

We generated five distinct network topologies. For each of these five network topologies, the simulation was independently executed five times using different random seeds for the background noise generation.

### Stimulation setup

To study responses to stimuli with varying regularity, we used an oddball paradigm with five stimuli (A–E), each delivered as a 1.0 ms current pulse (12 µA/cm²). Each label mapped to a unique spatially localized neuron pair within an annular region (2.0–3.0 mm from center). Only the ensemble neurons received stimulation. Each trial began with one such pulse. This stimulus amplitude ensured at least one spike for any $\:{g}_{Ks}$ value, providing a consistent activation baseline.

### Oddball paradigm

We ran three types of oddball sequences (200 trials each, ISI = 100 ms): (1) Standard: A appeared frequently (*p* = 0.8), B was rare (*p* = 0.2); (2) Deviant: B was frequent (*p* = 0.8), A was rare (*p* = 0.2); and (3) Control: All five stimuli appeared equally (*p* = 0.2), serving as a neutral baseline. Stimulus blocks (5-element sequences) were randomly concatenated to form each condition. Each network ran four A/B pairings (1/2, 2/1, 3/5, 5/3) and one control sequence, giving 20 datasets total.

### Simulation details and code

Simulations used custom MATLAB scripts with forward Euler integration (0.01 ms timestep). At each step, synaptic currents, state variable derivatives, and synaptic conductance updates were computed. Each experimental block (standard, deviant, control) began from identical resting states, ensuring comparable dynamics. Trials were 100 ms long. We logged spike times for all neurons and constructed PSTHs (1 ms bins, 40 ms window post-stimulus A) to compare population responses across contexts.

### Deviance detection

Our primary quantity of interest was the stimulus-evoked response magnitude, denoted as R. We defined R as the average number of spikes fired in response to a specific stimulus across all relevant trials. This measure captures the mean trial-wise response and can be calculated at different levels of granularity.

We used subscripts and superscripts to clarify the response context. For example, $\:{}^{seq}{R}_{p}^{S}$ denotes the response of neuron(s) p, averaged across all trials of type S within the stimulus sequence seq. The index p could refer to: a specific neuron (e.g., *i*, *j*), a subpopulation (e.g., exc), or the entire network (if omitted), in which case R represents the median response across neurons. The trial type S could take one of the following values: A or B: indicating trials where stimulus A or B was presented, N: indicating non-target trials (stimuli C, D, or E). We defined the contrast in response between two conditions using the notation

12 $$\:\varDelta\:R={}^{A-as-dev}R-{}^{A-in-con}R$$

When aggregating over a population p, we used $\:\stackrel{-}{{R}_{p}}$ to denote the mean of individual responses within that population.

To isolate genuine DD, we followed established protocols that avoid conflating deviance with mere stimulus identity or adaptation effects. Specifically, we did not compare stimulus A in the A-as-dev and A-as-std sequences, as this would conflate deviance with adaptation due to frequent presentation. Instead, we compared responses to stimulus A in the A-as-dev and A-in-con sequences. The A-in-con condition serves as a neutral control, where all stimuli are presented with equal probability (*p* = 0.2). Thus, stimulus A appears as infrequently as in the deviant condition but without the context of an expected standard stimulus B. This control allows us to attribute any increase in response solely to statistical deviance, rather than repetition effects or physical differences between stimuli. To quantify this effect, we defined the Deviance Detection Index (DDI) as:

13 $${\text{DDI }}=\left( {\overline {{{}_{{}}^{{A - as - dev}}{R^A} - \overline {{{}_{{}}^{{A - in - con}}{R^A}}} }} } \right)/\left( {\overline {{{}_{{}}^{{A - as - dev}}{R^A}}} +\overline {{{}_{{}}^{{A - in - con}}{R^A}}} } \right)$$

Here, $\:\stackrel{-}{{}^{A-as-dev}{R}^{A}}$ and $\:\stackrel{-}{{}^{A-in-con}{R}^{A}}$ represent the mean number of spikes fired per neuron in response to stimulus A under the deviant and control conditions, respectively. A higher DDI value indicates stronger sensitivity to statistical deviance, independent of adaptation or identity effects.

### Spike frequency adaptation

Spikes were defined as upward membrane crossings of 0 mV. Only spikes after 100 ms (post-stimulus) were analyzed. ISIs were calculated from spike time differences. SFA was quantified as:

14 $$\:\mathrm{S}\mathrm{F}\mathrm{A}\:\mathrm{I}\mathrm{n}\mathrm{d}\mathrm{e}\mathrm{x}=\frac{{ISI}_{last}-{ISI}_{first}}{{ISI}_{first}}$$

This index was computed only if ≥ 3 spikes occurred and $\:{ISI}_{last}$ ≥ I$\:{ISI}_{first}$. If spike rate increased ($\:{ISI}_{last}$< $\:{ISI}_{first}$), SFA was set to 0. Trials with < 3 spikes were excluded.

### Mean phase coherence (MPC)

To compute phase $\:{\varphi\:}_{ij}^{k}$ of spike *k* from neuron *i* relative to spikes of neuron *j*:

15 $$\:{\varphi\:}_{ij}^{k}=2\pi\:\frac{{t}_{k}^{i}-{t}_{-}^{j}}{{t}_{+}^{j}-{t}_{-}^{j}}$$

where $\:{t}_{k}^{i}$ and $\:{t}_{+}^{j}$ are the preceding and following spikes from neuron *j*, $\:{t}_{-}^{j}$ is the time of the last spike fired by neuron *j* before $\:{t}_{k}^{i}$. Pairwise coherence $\:{r}_{ij}$ was computed as the magnitude of the mean complex vector:

16 $$\:{r}_{ij}=\frac{1}{T}\sum\:_{k=0}^{T}{e}^{i{\varphi\:}_{ij}^{k}}$$

where T is the number of spikes fired by neuron *i* that are between a pair of spikes fired by neuron *j*. $\:{r}_{ij}$ ranges from 0 (no synchrony) to 1 (perfect locking). Network-wide MPC was defined as the mean $\:{r}_{ij}$ across all pairs where both neurons fired > 30 spikes during the simulation.

## Supplementary Information

Below is the link to the electronic supplementary material.

Supplementary Material 1

## Data Availability

All data used to produce the figureswas generated via numerical simulations of the code, which is publicly available in the Zenodo repository under the DOI: 10.5281/zenodo.17072341.

## Code availability

The source code for our computational model and analysis scripts is publicly available in the Zenodo repository under the DOI: 10.5281/zenodo.17072341.
