# Supplementary material for: Acetylcholine enhances deviance detection in Hodgkin-Huxley neuronal networks
Source: Cogn Neurodyn. 2026 Aug 7;20(1):153. doi: 10.1007/s11571-026-10522-3 (PMC13451467; doi:10.1007/s11571-026-10522-3)
Supplement: Supplementary file 1 [file 11571_2026_10522_MOESM1_ESM.docx]

**Supplementary Information**

**Figure S1. Evoked response under strong current.** Change in membrane potential after an external stimulus current of 25 $\mu A/{cm}^{2}$ under $g_{Ks}$ = 0.1 and 1.5.

**Figure S2.** **The f/I curves with different** $\boldsymbol{g}_{\boldsymbol{Ks}}$ **values**. Different colors represent the different $g_{Ks}$ values (shown as the color bar).

**Figure S3. The phase-response** **curves with different** $\boldsymbol{g}_{\boldsymbol{Ks}}$ **values.** (**A**) The phase response curve is measured by comparing perturbed vs. unperturbed periods when neurons fire at a fixed frequency. When the next spike is earlier the phase response is positive (blue), when it is delayed it is negative (red). (**B**) Type 1 neurons have a strictly positive phase response curve (low $g_{Ks}$), while Type 2 neurons have a biphasic phase response curve (high $g_{Ks}\boldsymbol{)}$.

**Figure S4.** **DD in the sample network under varying** $g_{Ks}$ **levels.** (**A**) Network responses to the target stimulus A under three sequence types. A: std, A: dev, and A: con represent the evoked responses to stimulus A under the A-as-std (160 trials), A-as-dev (40 trials), and A-as-con (40 trials) sequences, respectively. Color indicates the total spike count across all neurons per millisecond. (**B**) Post-stimulus time histograms for network responses to stimulus A under the three sequence types. Sixteen $g_{Ks}$ conditions (1.5, 1.4, 1.3, 1.2, 1.1, 1.0, 0.9, 0.8, 0.7, 0.6, 0.5, 0.4, 0.3, 0.2, 0.1, and 0) are shown.
